# 

**Published:** 1911-01-14

**Authors:** 


**Pneumonia in Children.**

The onset of pneumonia in childhood, though typically abrupt, is frequently preceded by indefinite illness, often gastro-intestinal, which may be protracted over some weeks. The pneumonia comes as a final crash. This is most commonly met with during an epidemic of influenza. On no account in desperate cases, if you thoroughly understand the condition, worry about examining the chest. If the pneumonia is spreading it will probably mean death, and if it is not spreading the child wants all its strength for breathing and feeding.—*Dr. F. J. Poynton.*

**Muscular Power in Chorea.**

A very important feature in some cases of chorea is the loss of muscular power. Cases are sometimes diagnosed at first as paraplegia. The movements are often very trifling indeed, and may be only noticed after an interval. The most obvious symptom is the loss of motor power, and that must be carefully distinguished from the apparent loss of power which is present in every case of motor chorea. The disadvantage under which the muscular movements are carried out is obvious if the child is told to grip the hand; the inco-ordinate movements put the child at a mechanical disadvantage, and so one notices an apparent weakness which is not really due to a loss of muscular power.—*Dr. A. E. Garrod.*

### BOOKS RECEIVED.

Henry Frowde and Hodder and Stoughton.

"Manual of Practical Anatomy." Vols. I. and II. By A. Robinson.

"Outlines of Zoology." Fifth edition. By J. A. Thompson.

Scientific Press, Ltd.

"Gynæcology for Nurses and Gynæcological Nursing." By Comyns Berkeley.

"Massage Movements." New edition. Revised by Miss Atkey.

"Surgical Instruments and Appliances." New edition. By H. Burrows.

Baillière, Tindall and Cox.

"Some Considerations of Medical Education." By S. Squire Sprigge.

"On Acute Intestinal Toxæmia in Infants." By R. Vincent.

"Pupil Midwives' Register of Cases." By G. C. Marks.

Longmans, Green.

"Essentials of Histology." By Schäfer.

"Notes on Physiology." By Ashby.

"Urinary Surgery." By J. S. Kidd.

"Practical Motherhood." By Campbell.

Wm. Green and Sons.

"Manual of Diseases of Women." By Fothergill.

J. and A. Churchill.

"Hernia: Its Treatment." By R. W. Murray.

"Taylor's Principles and Practice of Medical Jurisprudence." Vols. I. and II. Sixth edition. By F. J. Smith.

"Pharmacology and Therapeutics, or the Action of Drugs." By A. R. Cushny. Fifth edition.

### Gifts and Bequests.

Mr. Joseph Lees, of Smith Street, Oldham, Lancs, builder, has bequeathed £100 to the Oldham Infirmary.

Miss Irvina Smale, of Ladbroke Terrace, Notting Hill, W., has bequeathed £500 to the Great Northern Central Hospital.

Miss Anna Maria Crabbe, of Norfolk Road, Brighton, has bequeathed £100 each to the Sussex County Hospital and to the Sussex County Homœopathic Dispensary. She desired that the charities should be recorded in the books of the various societies as "Anonymous."

Mrs. Mary Elizabeth Baker, of Castle Wiveliscombe, Somerset, sister of the late Mr. Clement Scott, bequeathed £10,000 each to the Bristol General Hospital, the Lying-in ward in connection with the Proctor Baker ward, and the Hospital for Consumptives, Winsley, Wilts, unless she shall have given these sums during her lifetime.

Miss Henrietta Louisa Maw, of Ivy House, Withington, Manchester, bequeathed £1,000 each to the Manchester Royal Infirmary to endow a bed there in perpetuity, directing that a tablet should be placed at the head of that bed recording the fact that it was endowed by her, and the Lincoln County Hospital for the endowment of a bed there in perpetuity, desiring that as far as possible it may be used for the inhabitants of Epworth.

Mr. Samuel Staniforth, of Sheffield, blade manufacturer, has bequeathed £1,000 each to the Sheffield Royal Infirmary and the Sheffield Royal Hospital; £300 to the Jessop Hospital for Women, Sheffield; and £200 to the Sheffield Children's Hospital

Mr. Frederick Platt-Higgins, of Woodham Place, Horsell, Woking, cotton spinner, Conservative M.P. for North Salford, 1895-1906, bequeathed £250 to the Hospital for Consumption and Diseases of the Throat and Chest.

Mr. William D'Amery Amery, of The Manor House, Eckington, Worcestershire, has bequeathed £100 to the Pershore Cottage Hospital.

Prince Alexander of Teck has received the following further contributions to the Prince Francis of Teck Memorial Fund for the endowment of the Middlesex Hospital: Mr. Burdett-Coutts, M.P., £52 10s.; Lady Waterlow and Mrs. John Mackinnon, £50 each; Mr. George P. Gooch, £25; Mr. S. H. Whitbread, £10.

Mr. Henry Elliot Tracey-Elliot, of St. James's Terrace, Plymouth, and of Leigham, Eggbuckland, Devon, who left estate valued at £158,118 11s. gross, has bequeathed £3,000 to the South Devon and East Cornwall Hospital, Plymouth, £2,000 to the Plymouth Public Dispensary, and £2,000 to the Plymouth Royal Eye Infirmary. These bequests in each case are to be added to capital and not treated as income.

Sir Charles Scotter, first baronet, of Rutland House, Kingston-on-Thames, chairman of the London and South Western Railway Company, and formerly general manager of that company, who left estate of the gross value of £61,565, has left £100 each to the Kingston Cottage Hospital, the Surbiton Cottage Hospital, and the Royal London Ophthalmic Hospital.

The following charitable grants have been made by the Tallow Chandlers' Company in the present month: London Hospital, £21; East London Hospital for Children, Middlesex Hospital, Royal Waterloo Hospital for Children and Women, Princess Mary's Convalescent Home (Bognor), Guy's Hospital, Royal Hospital for Incurables (Putney), St. Bartholomew's Hospital, and Great Northern Central Hospital, £10 10s. each; Eastbourne Convalescent Home and Seaford Convalescent Home, £5 5s. each.

### Jottings.

The King and Queen have sent pheasants and other game to the West Norfolk and Lynn Hospital.

Princess Henry of Battenburg sent a decorated miniature Christmas-tree for the annual treat given at the Belgrave Hospital for Children last week.

Two performances of "Caste" were given in aid of the Horsham branch of the League of Mercy at the Roffey Institute, Horsham, on Wednesday, January 11, at 8 p.m., and Thursday, January 12, at 2.45 p.m.

Queen Alexandra sent a present of sweets for the annual Christmas treat of the patients in the Alexandra Hospital for Children with Hip Disease, Queen Square, Bloomsbury, of which her Majesty is patron. Sixty-eight children were entertained at the treat.

The Grantham Hospital Ball, which also served as the annual dance for the members of the Belvoir, took place last week in the Guildhall, Grantham, under the patronage of Countess Brownlow. The company included many members of the Belvoir, Cottesmore, and Blankney Hunts.

At the New Year entertainment at the Great Northern Hospital, on Tuesday last, Mr. Frederick Hammand, Chairman of the House Committee, stated that the patients had to thank the King for the pleasure they had had, because he suggested it in a charming letter to the hospital.

At Plymouth last week, the Mayor presiding, it was decided to raise as a memorial to King Edward a fund for affording treatment to persons suffering from consumption, to be called the King Edward Fund. The interest will be devoted to measures for its prevention and to treatment in sanatoria.

A correspondent has received the following letter from Sir Arthur Bigge: "York Cottage, Sandringham, January 5, 1911.—Sir, I am commanded to thank you for your letter of the 2nd inst., in which you suggest that the issue of a special Coronation stamp might be a source of income to the King's Hospital Fund, and, in reply, his Majesty directs me to inform you that this matter has been under consideration."

The Bishop of Chichester has written a letter to the *Times*, from which we take the following: During a short stay in Nazareth last month I found my way to the hospital there belonging to the Edinburgh Medical Missionary Society. I found a splendid work being done in a house that is rented temporarily, and a large number of surgical cases, under the care of one doctor and two trained nurses. When I say that in one year 677 important operations have been performed, that there have been 13,734 attendances at the hospital, and 1,224 visits to patients in their own homes, some faint idea of the magnitude of the work that is being done by these three workers may be formed. . . . in premises ill-adapted for such a purpose and terribly understaffed. A call for help that comes from Nazareth at Christmastide. Dr. Sargood Fry is the secretary of the society, and its headquarters are 55 George Square, Edinburgh.

### TO HOSPITAL SECRETARIES AND OTHERS.

We invite contributions from any of our readers, and shall be glad to pay, according to length, for items of news for the institutional section (including appointments, resignations, gifts, etc.) which we may publish. All payments for manuscripts used are made as early as possible after the beginning of each quarter.

### THE HOSPITAL WORLD.

### THE MEDICAL SCHOOLS.

The following are the arrangements for next week at the Medical Graduates' College and Polyclinic, 22 Chenies Street, W.C. : Monday, January 16, at 4 p.m., Dr. T. Colcott Fox, Clinique (Skin); at 5.15 p.m., Dr. Theodore Thompson, "Anæsthesia as an Aid in Diagnosis." Tuesday, at 4 p.m., Dr. G. Newton Pitt, Clinique (Med.); at 5.15 p.m., Dr. Nathan Raw (Liverpool), "Modern Methods of Treatment of Tuberculosis." Wednesday, at 4 p.m., Mr. T. P. Legg, Clinique (Surg.); at 5.15 p.m., Mr. Angus McNab, "Myopia." Thursday, at 4 p.m., Mr. A. Carless, Clinique (Surg.); at 5.15 p.m., Mr. Jackson Clarke, "Treatment of Certain Common Fractures." Friday, at 4 p.m., Mr. R. E. Bickerton, Clinique (Eye).

The following are the arrangements for next week at the West London Hospital, Hammersmith Road : At 10 a.m., January 16 and 19, demonstration by Surgical Registrar; at 10 a.m., January 18, gynæcological demonstration; at 11.30 a.m., January 17, demonstration of minor operations; at 12 noon, January 16, pathological demonstration; and at 12 noon, January 19, lecture, Dr. Grainger Stewart, Practical Medicine. Lectures at 5 p.m. will be given on January 16, by Mr. Armour, on "Drainage in Abdominal Surgery"; January 17, Dr. Davis, on Throat and Ear Diseases, Clinical Lecture I.; January 18, Dr. Beddard, on Practical Medicine, Lecture I.; January 19, Dr. Davis, on Throat and Ear Diseases, Clinical Lecture II.; and on January 20, Dr. Saunders, Clinical Lecture, with Cases.

### OBITUARY.

At the age of seventy-eight, at Richmond, Surrey, the death has taken place of Charles Henry Johnson, J.P., M.R.C.S., L.S.A., Staff Surgeon and Captain to the Turkish Contingent, Crimea. He had received the Order of the Medjidieh.

The death is reported at Sketty, Glamorganshire, of Mr. Ebenezer Davies, M.R.C.S., at the age of eighty-one. Mr. Davies took his L.S.A. in 1852, and became an M.R.C.S. in the same year. He held the post of medical officer of health for Swansea for forty-three years.

The death is announced of Colonel Francis Howard, late of the Army Medical Staff, at Bournemouth. Colonel Howard was educated at Ledwich School, took his L.R.C.S. Ireland in 1861, his L.M. in 1862, when he obtained the M.D. degree of the University of St. Andrews. He saw service in the Afghan war of 1878, being awarded the medal. He was also with the Egyptian expedition of 1882, and received the medal and bronze star. He was formerly senior medical recruiting officer for the London district. He retired in 1893, but was re-employed at the War Office during the South African war and promoted to the rank of colonel in 1902. He was the author of a handbook of medical organisations of foreign armies.

### THE WEEK'S NOVELTIES.

**MESSRS. WELFORD AND SONS, LTD.,** of Elgin Avenue, Maida Vale, W., have received a Royal Warrant of Appointment as "purveyors of milk and cream" to his Majesty King George V.

**OXO** has now won such an established reputation that no medical man is at all likely to be unacquainted with it. At the same time it is worth while drawing attention to the great care which is taken in preparing this popular food and stimulant. Oxo is only made from guaranteed fresh and healthy beef, and the purity of the concentrated fluid is thus above suspicion.

"Blind Man's Buff," by Arthur Elsley, is the title of a handsomely framed photo-engraving which **BOVRIL, LIMITED,** has issued recently in connection with a remarkable prize scheme. Two other striking gravures—"Babes in the Wood," by F. Morgan, and "Tales of the Deep," by Joseph Clark—are also included in the scheme, full particulars of which will be forwarded on application to the Company's head office, 152 Old Street, E.C.

The Aylesbury Dairy Company has taken much trouble in the search for a concentrated milk, which could be mentioned seriously as a substitute for human milk in cases where for one reason or another that was not available. **HUMANOID** represents the result of their endeavour, and is a concentrated humanised milk specially prepared for the Aylesbury Dairy Company, and which needs only dilution with water. The medical profession can receive a free sample on writing to the chief office, 31 St. Petersburgh Place, Bayswater.

The value of a good tonic wine which combines the virtues of a fine vintage with added nutrition and absence of nervous stimulant is apparent even to the layman. **WINCARNIS,** as manufactured by Coleman & Co. at the company's Norwich factory, has long held a high place in public and professional esteem. Any practitioner, however, who wishes himself to test its quality has only to send for a trial bottle, which will be packed free to him on accompanying his request by his card and the mention of The Hospital.

It is not always that a new labour-saving appliance is accompanied by more perfect sanitation, but these two virtues are to be found in the vacuum cleaner system, which gets rid of elbow grease and germs at the same time. The **DAISY VACUUM CLEANER** is the best example of this type of cleanser, and its powerful suction removes all foreign matter from the objects to which it is applied. Model B is advertised at 7 guineas, and may be obtained direct from the Daisy Vacuum Cleaner Co., Gravelly Hill, Birmingham.

[Further particulars of these preparations are given in our advertisement columns this week.]

JUST PUBLISHED. Price **3s. 6d.** net. Cloth, 8vo. pp. ix, 204.

**THE DAWN OF THE HEALTH AGE**

**By BENJAMIN MOORE.**

A book dealing with medical reorganisation, hospital reform, and other medico-social problems of the hour.

London: J. & A. CHURCHILL, 7 Great Marlborough Street.
Liverpool: THE LIVERPOOL BOOKSELLERS CO., 70 Lord Street.

